# Artisanal shark fishing in Milne Bay Province, Papua New Guinea: biomass estimation from genetically identified shark and ray fins

**DOI:** 10.1038/s41598-018-25101-8

**Published:** 2018-04-27

**Authors:** S. A. Appleyard, W. T. White, S. Vieira, B. Sabub

**Affiliations:** 1 CSIRO Australian National Fish Collection, National Research Collections Australia, Castray Esplanade, Hobart, 7001 Tasmania Australia; 2 CSIRO Oceans & Atmosphere, Castray Esplanade, Hobart, 7001 Tasmania Australia; 3 doMar Research, P.O. Box 223, Fremantle, 6959 Western Australia Australia; 4 Papua New Guinea National Fisheries Authority, P.O Box 2016, Port Moresby, National Capital District Papua, New Guinea, Australia

**Keywords:** DNA sequencing, Ichthyology

## Abstract

Our study is the first detailed examination of species composition using DNA COI barcoding of elasmobranchs from an artisanal fishery of Papua New Guinea. The study is the first in the region to provide biomass estimates based on species confirmation following examination of dried fins. Over 20 species of elasmobranchs were identified from 623 fins from the artisanal fishery in Milne Bay Province of PNG, with *Carcharhinus amblyrhynchos* and *Carcharhinus melanopterus* the most abundant species in the catches. Of concern, 21% of fins examined were from IUCN listed threatened species (Vulnerable or Endangered) with 8% of fins from the Endangered scalloped hammerhead (*Sphyrna lewini*). Following species identifications and use of species-specific length and weight extrapolations, we estimated over 9 t of elasmobranchs contributed to the fin batch. Importantly, the vast majority of the elasmobranchs in this batch were from immature animals. Genetic identification has an important role to play in the ongoing sustainable management of elasmobranchs in artisanal fisheries in PNG and more widely. However in the absence of ongoing genetic testing, recording the species (if known) at the time of catch is more achievable and would provide more robust data for fisheries managers in PNG over the longer term.

## Introduction

As apex predators which serve important and unique roles in the marine ecosystem^1–3^, many elasmobranch species (i.e. Class Chondrichthyes, sharks and rays) and populations are under significant pressure from fisheries-driven declines^4^. Much of this decline is linked to the demand for shark fins^5–10^. Shark fin imports to Asia have been reported at up to 20 000 t per year^11^ with Hong Kong previously considered the global centre of shark fin trade^5,9,10^. Recently, Hong Kong was surpassed by Thailand (from 2007 to 2011) as the world’s largest exporter of shark fins^12^ and currently, Indonesia is now considered the world’s largest shark producer. Despite the increase in shark products, there is limited capacity to assess the sustainability of the shark landings and species compositions in these fin products^8,9,13^. This shark fin demand, coupled with the intrinsic biological attributes of some sharks and rays (i.e., including slow growth rates, low fecundity, late maturity and long gestation leading to relative lower productivity)^5,14,15^ and increasingly high prices being paid for fins^16^ puts significant pressure on elasmobranch species. As a result, a number of elasmobranch species are now subject to international trade restrictions under the Convention on International Trade in Endangered Species of Wild Fauna and Flora (CITES)^17,18^ (as at https://www.cites.org/eng/app/appendices.php, 28 March 2018). As of 2017, the sawfishes (Family Pristidae) are listed on CITES Appendix I, and 12 other elasmobranch species are listed in CITES Appendix II. Additionally, regional fisheries management organisations (e.g., Western & Central Pacific Fisheries Commission (WCPFC)) recently established conservation and management measures for several shark species, e.g., silky sharks *Carcharhinus falciformis* (https://www.wcpfc.int/doc/cmm-2013-08/conservation-and-management-measure-silky-sharks).

World-wide, stock assessments of elasmobranch species are severely hampered by the lack of species specific catch and trade data^3,5,6,12,19^ compounded further by catches of sharks from illegal, unregulated and unreported (IUU) fisheries^5,20^. In developing countries, the stock assessments of elasmobranchs is further exacerbated when accurate morphological and species identifications of individuals (and shark products) are not attainable. Insufficient monitoring of landings, and fisheries (that are not well regulated) that target elasmobranch species, results in unknown or highly underestimated rates in west Pacific and Pacific countries^16,21^. The shark fin industry further compounds these aspects as most detached dried fins are difficult to identify to species level, they often lack diagnostic features^5,8,12,22^ and there is generally no information kept with regards to the harvested species (even if known).

In the west Pacific country of Papua New Guinea (PNG), limited government resourcing and relatively large fishing territories, coupled with lack of species identification tools for elasmobranchs makes it challenging to quantify shark landings and fin catches more explicitly^16^. Furthermore, the combination of various shark fishing activities, including both managed larger scale fisheries (where sharks are taken as bycatch^23,24^) and localised, coastal (herein referred to as artisanal) small scale fishing activities that use small vessels, are largely unmanaged and are not well understood^16^) makes it difficult to quantify shark landings. The management of large scale shark fishing in PNG was previously governed by the National Shark Longline Management Plan, however following the WCPFC 2014 ban on the retention of silky shark, the shark fishery ceased operating^16^. In contrast, there are currently no national management arrangements in place for the artisanal fishery^16^.

While FAO statistics demonstrate that PNG shark harvests are low when compared to other countries’ estimates, based on official in-country data (informed by Local Level Government (LLG) information which provides some indication of locality), harvests are likely to be underestimated with shark fishing increasing dramatically over the last three decades^16,24,25^. Adding to this^16^, while several shark species are vulnerable in PNG, local fishers also depend on shark fin for income. Information from the artisanal sector suggests that the Milne Bay Province of PNG, which is at the south eastern tip of mainland PNG, is a focal point for artisanal shark fishing activities^16^ (although the province is not known as a nursery area). Artisanal fishers in the area are based primarily at the Louisiade Rural LLG of the Milne Bay Province and while species catches from this region of PNG have not previously been analysed in any detail, relatively high quantities of dried fins have been noted from this Province (i.e., between 2.1 t and 3.9 t per year in 2010–2014)^16^. Despite this, no national management arrangements exist for the capture and utilisation of shark in the province^16^. An opportunity therefore exists to provide better informed baseline data on the species compositions and catches of elasmobranch harvests in the Milne Bay Province through the application of accurate species identification and delineation of fins (from the artisanal fishery) to provincial shark fin buyers.

There are a number of tools and classification categories that can be used to help identify the species of origin of shark fins. In the Hong Kong and China markets, shark fins are delineated on the quality of the fin rays/needles and through distinguishing features of the dried fin (with up to eleven market categories in place for describing shark fins^5,12^). However, these product categories are market specific and not generally applicable for taxonomic species identifications or determination of species compositions. Stable isotope, infrared spectroscopy and electron microscopy analyses have also been used to examine the authenticity of dried shark fins (i.e., real dried fins, fake dried fins and artificially dried fins) although the species of origin of the fins cannot be determined with these methods^26^. There are now also various qualitative and analytical tools described in the literature^12,22^ and from online websites (https://cites.org/eng/node/16695; https://cites.org/eng/prog/shark/isharkfin; http://www.sharkfinid.com/p/online-identification.html) that can be used for the morphological/taxonomic identification of shark fins. Nonetheless, in our PNG study, we found that these analytical tools were not 100% reliable for taxonomic species identifications, were not ideal or suitable for our use in some field circumstances and the morphological identification tools are often limited to certain species. We therefore infer the most accurate and robust method for species identification of elasmobranch fins (irrespective of source location) is DNA barcoding.

DNA barcoding of shark fins and/or the use of species-specific PCR primers for shark species identifications has been undertaken previously^3,5,8,27^. Barcoding utilises a highly reproducible automated DNA based identification method^28–33^ which sequences the nucleotide composition of relatively short mitochondrial DNA (mtDNA) fragments. Typically, the cytochrome c oxidase subunit I (COI) gene (and other genes such as 16 S ribosomal RNA (16 S rRNA); mtDNA encoded NADH dehydrogenase 2 (ND2)) are used. Success depends on within species DNA sequences being more similar to those between species^31^. By matching a COI barcode sequence from a fin clip against a reference library (e.g., public repository of the Consortium for the Barcode of Life (BOLD, www.boldsystems.org32)), we can determine the shark species from which the fin was taken (based on low intra- but high inter-species diversity^28^). Given that several shark species have similar fins with respect to morphology, colour and size, DNA barcoding provides us with the only means to accurately identify the constituent species.

Accurate species identification of elasmobranch fins enables us to understand which species are being caught in the artisanal fishery in the Milne Bay Province and to what extent, as well as improving our knowledge of the biodiversity in the region. As part of a Commonwealth Scientific and Industrial Research Organisation (CSIRO)/Australian Centre for International Agricultural Research (ACIAR)/Papua New Guinea National Fisheries Authority (PNG NFA) research, we are utilising molecular technologies to provide fisheries managers with biodiversity information (and in many instances, baseline biological data) on various shark and ray species in PNG. In the CSIRO/ACIAR/PNG NFA research, we developed a barcode library for known elasmobranch species in the region. Herein we use this library to accurately identify the genetic species composition of elasmobranch fins from the artisanal fishery in the Milne Bay Province of PNG. While a number of recent shark fin papers have examined species characterisation and distribution in the region (e.g. from illegal fishing in Australian waters^27^; the Indonesian shark fishery^8^; Taiwan’s ports, markets and customs detention^3,19^), this is the first barcoding study of any kind in PNG. This study is also one of the first to utilise genetic species identifications of fins to extrapolate to elasmobranch catch/biomass from an artisanal fishery.

## Results

Out of the 623 individuals recorded in the artisanal sourced batch of fins from Asiapac (one of two licensed fish buyers in the Milne Bay Province), 557 fin samples were extracted and genetically analysed. The other 66 fins were identified to species based on morphology (from images) and colouration. While we ran some of our shark fin images through the iSharkFin application (https://cites.org/eng/prog/shark/isharkfin), we were not able to generate reliable species identifications and deemed it not fit for purpose for the PNG fins. Thus genetic species identification was undertaken on the bulk of the fins.

### Genetic identifications

Amplification at the COI gene in 557 fins resulted in 55 samples not amplifying successfully in the first instance. As we were sequencing 96 samples per plate, DNAs had been normalised and we were aiming for moderate-throughput identifications, it was not cost effective to ‘cherry pick’ or repeat samples that did not amplify in the first instance. We did not further troubleshoot the DNA or PCR amplifications of those samples. Based on re-examination of images with subsequent genetic identifications obtained (see below), all but 7 of the 55 samples that did not amplify could be identified to species level; the remaining 7 were only identified as belonging to the family Carcharhinidae.

Following consensus sequence generation, BLAST comparisons and sequence quality control, elasmobranch species identifications based on COI sequences were obtained for 470 fins. In total, 22 species across eight genera and six families (Hemigaleidae, Carcharhinidae, Sphyrnidae, Pristidae, Rhinidae and Glaucostegidae) were genetically identified from the fins. Nineteen shark species and three ray species were recorded (Table 1). The Jukes-Cantor distance among the 22 species observed within PNG regional waters was 0.061 (se = 0.006), while the within species distance ranged from 0.000 ± 0.000 (e.g., *Galeocerdo cuvier*, *Carcharhinus sorrah*) to 0.009 ± 0.002 (*Negaprion acutidens*) (see Table 1).

**Table 1:** Species of sharks and rays genetically identified from dried PNG shark fins including: genetic sample size (number of individuals’ barcoded), COI genetic information (nucleotide composition, fragment length and GenBank information) and IUCN species status

Species^IUCN status*^	Genetic sample size	COI	nucleotide	composition^**^			Average divergence within species ( ± se)		Representative GenBank Accession Numbers
		T	C	A	G	bp			
*Hemipristis elongata*^VU^	2	35.7	23.8	24.8	15.7	587.5	0.002 (0.002)	MF508658, MF508659	
*Carcharhinus albimarginatus*^VU^	20	34.9	23.5	26.6	15.1	631	0.000 (0.000)	MF508660	
*Carcharhinus altimus*^DD^	2	35.7	22.6	26.9	14.7	631	0.002 (0.002)	MF508661, MF508662	
*Carcharhinus amblyrhynchoides*^NT^	15	35.8	22.7	26.5	15	630.9	0.001 (0.000)	MF508663, MF508664	
*Carcharhinus amblyrhynchos*^NT^	219	36.4	22	26.6	14.9	618.4	0.002 (0.001)	MF508665, MF508666, MF508667, MF508668, MF508669, MF508670	
*Carcharhinus amboinensis*^DD^	3	35.2	23.5	26.8	14.5	573.7	0.001 (0.001)	MF508671, MF508672	
*Carcharhinus brevipinna*^NT^	5	34.2	24.6	26.2	15	630.8	0.000 (0.000)	MF508673	
*Carcharhinus falciformis*^NT^	19	35.8	22.7	26.3	15.2	631	0.002 (0.001)	MF508674, MF508675, MF508676, MF508677	
*Carcharhinus leucas*^NT^	6	35.4	23.4	26.6	15.1	630.9	0.001 (0.001)	MF508678, MF508679	
*Carcharhinus limbatus*^NT^	34	35.7	22.8	26.3	15.2	630.9	0.000 (0.000)	MF508680	
*Carcharhinus melanopterus*^NT^	13	34.8	23.5	26.6	15.1	630.9	0.000 (0.000)	MF508681	
*Carcharhinus plumbeus*^VU^	2	35.5	22.8	27	14.7	631	0.000 (0.000)	MF508682	
*Carcharhinus sorrah*^NT^	18	35.3	23	26.8	14.9	631	0.000 (0.000)	MF508683	
*Carcharhinus tilstoni*^LC^	18	36	22.5	26.4	15.1	631	0.000 (0.000)	MF508684	
*Galeocerdo cuvier*^NT^	22	34.9	23.1	27.1	14.9	631	0.000 (0.000)	MF508685	
*Negaprion acutidens*^VU^	22	35.3	23.3	25.8	15.5	631	0.009 (0.002)	MF508686, MF508687	
*Sphyrna lewini*^EN^	32	33.7	24.5	26.3	15.5	631	0.003 (0.001)	MF508688, MF508689, MF508690, MF508691, MF508692	
*Sphyrna mokarran*^EN^	6	33.9	24.4	26.8	14.9	631	0.000 (0.000)	MF508693	
*Sphyrna zygaena*^VU^	4	34.5	23.6	26.5	15.4	631	0.000 (0.000)	MF508694	
*Anoxypristis cuspidata*^EN^	2	31.9	26.3	26.3	15.5	631	0.000 (0.000)	MF508695	
*Rhynchobatus australiae*^VU^	2	31.9	22.8	26.5	15.1	631	0.000 (0.000)	MF508696	
*Glaucostegus typus*^VU^	5	33.1	24.7	24.8	17.3	631	0.003 (0.001)	MF508697, MF508698	
Average	21.4	34.8	23.5	26.4	15.2	625.8			

^*^as at 27 April 2017, www.iucnredlist.org, vers 3.1 IUCN 2001, categories Near Threatened (NT), Vulnerable (VU), Endangered (EN); Data Deficient (DD); Least Concern (LC); **COI sequence lengths ranged from 574 bp to 631 bp with an average of 625.8 bp with an average nucleotide composition of T: 34.8%, C: 23.5%, A: 26.4% and G: 15.2%.

There was a low level of genetic divergence observed in the Hemigaleidae, Carcharhinidae, Pristidae and Rhinidae individuals (0.000–0.002) while for the Sphyrnidae, only fins from *S. lewini* showed genetic variation (genetic divergence = 0.003). There were no genetic differences detected at the COI fragment screened in the *Sphyrna mokarran* or *Sphyrna zygaena* individuals in this study. The five Glaucostegidae individuals also showed genetic divergence (0.003).

### Species compositions

**Figure 1:**
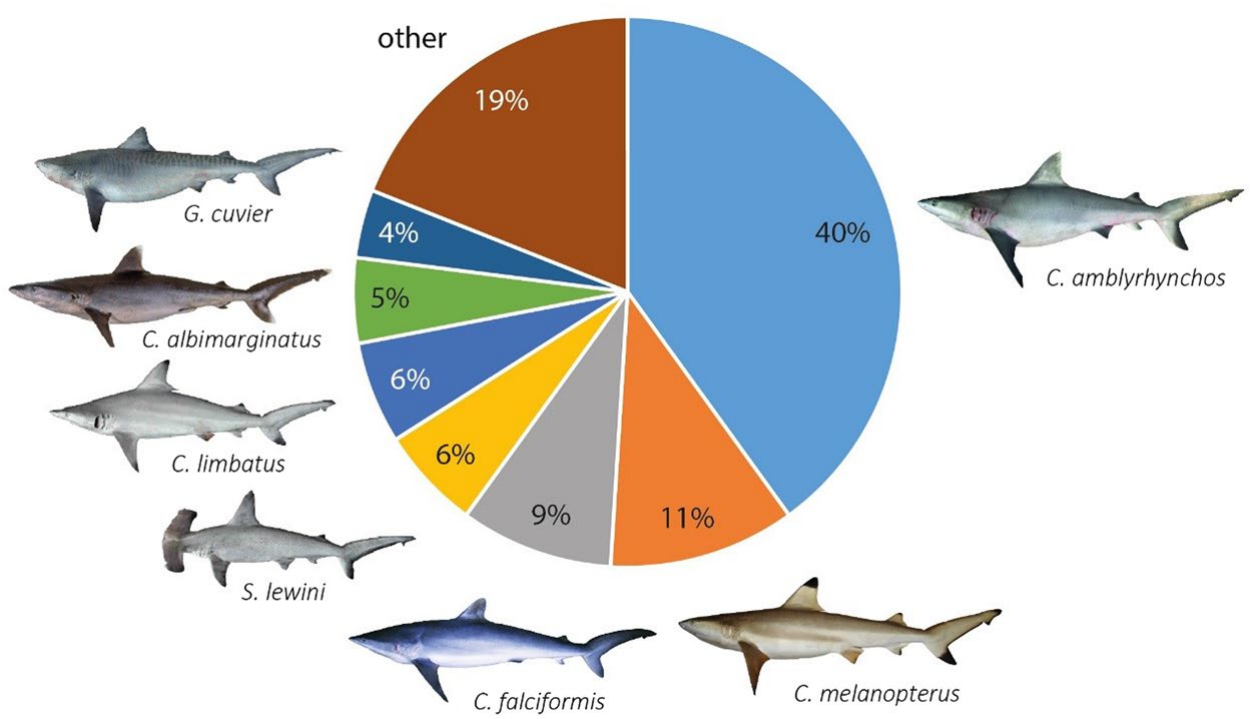
Composition of the most abundant species (i.e., number of individuals) as identified from the dried shark fins following morphological and genetic species identification (images ©Australian National Fish Collection).

Of the 22 species, the most commonly observed species (based on number of individuals) represented in the full sample of fins was the grey reef shark (*C. amblyrhynchos*) followed by blacktip reef shark (*C. melanopterus*), silky shark (*C. falciformis*), scalloped hammerhead (*S. lewini*) and blacktip shark (*Carcharhinus limbatus*) (Fig. 1). Fins from the smooth hammerhead shark (*S. zygaena*), narrow sawfish (*Anoxypristis cuspidata*) and several other carcharhinid species (e.g., *Carcharhinus altimus* and *Carcharhinus plumbeus*) were rarely observed. Individuals from species in the IUCN threatened categories (VU, EN and CR) accounted for 21% of the genetically identified dried shark fins. The endangered hammerhead species (*S. lewini* and *S. mokarran*) accounted for approximately 8% of the fins.

### Total catch estimations based on fins

Following species assignment of fins, the fin measurements were used to estimate total lengths and weights for each individual (see Table 2). The estimated total length of elasmobranchs from which fins had been taken ranged from 40–325 cm representing an extrapolated total catch of over 9 t (as estimated from this fin batch). Estimated biomass per species that contributed to this batch of 150 kg of dried fins from the Milne Bay Province, ranged from 11 kg of *Rhynchobatus australiae* to 2 323 kg of *C. amblyrhynchos*. Additionally, we estimated over 1 000 kg of IUCN listed Endangered shark species have recently been taken out of the artisanal waters in the Milne Bay Province, with over 10% of this shark biomass coming from hammerhead sharks.

**Table 2:** Species of sharks and rays identified from the dried shark fins including: number of individuals, ranges of estimated total length and estimated total individual weight, and total estimated weight.

Family	Scientific name		Estimated length (cm)		Estimated weight (kg)		
Common name		#	Min.	Max.	Min.	Max.	Total
Hemigaleidae							
Fossil Shark	*Hemipristis elongata*	2	102	184	4.5	30.3	34.8
Carcharhinidae							
Silvertip Shark	*Carcharhinus albimarginatus*	28	69	201	1.8	55.3	328.3
Bignose Shark	*Carcharhinus altimus*	2	169	190	29.5	43.0	72.5
Graceful Shark	*Carcharhinus amblyrhynchoides*	16	52	137	0.8	19.1	137.4
Grey Reef Shark	*Carcharhinus amblyrhynchos*	251	40	141	0.4	18.9	2322.7
Pigeye Shark	*Carcharhinus amboinensis*	5	79	220	3.1	88.2	237.0
Spinner Shark	*Carcharhinus brevipinna*	6	78	194	2.2	46.6	119.8
Silky Shark	*Carcharhinus falciformis*	54	96	303	5.1	207.3	2093.7
Bull Shark	*Carcharhinus leucas*	9	101	230	7.0	98.4	459.1
Common Blacktip Shark	*Carcharhinus limbatus*	39	66	192	1.2	34.3	224.9
Blacktip Reef Shark	*Carcharhinus melanopterus*	69	70	121	1.7	12.8	380.4
Sandbar Shark	*Carcharhinus plumbeus*	3	156	177	25.8	39.2	92.1
Spottail Shark	*Carcharhinus sorrah*	18	69	119	1.9	11.8	78.1
Australian Blacktip Shark	*Carcharhinus tilstoni*	17	51	141	0.8	17.8	139.3
Tiger Shark	*Galeocerdo cuvier*	24	98	306	3.9	159.5	976.2
Sicklefin Lemon Shark	*Negaprion acutidens*	14	59	150	0.8	17.3	118.8
unknown carcharhinid		7	86	113	3.8	9.7	50.7
Sphyrnidae							
Scalloped Hammerhead	*Sphyrna lewini*	40	76	242	2.0	67.0	756.0
Great Hammerhead	*Sphyrna mokarran*	6	128	235	8.3	59.1	165.2
Smooth Hammerhead	*Sphyrna zygaena*	4	122	152	9.2	17.0	48.2
Pristidae							
Narrow Sawfish	*Anoxypristis cuspidata*	1	325	325	81.7	81.7	81.7
Rhinidae							
Whitespotted Wedgefish	*Rhynchobatus australiae*	1	136	136	10.7	10.7	10.7
Glaucostegidae							
Giant Guitarfish	*Glaucostegus typus*	7	97	205	3.8	33.5	167.1
TOTAL		623					9094.8

**Figure 2:**
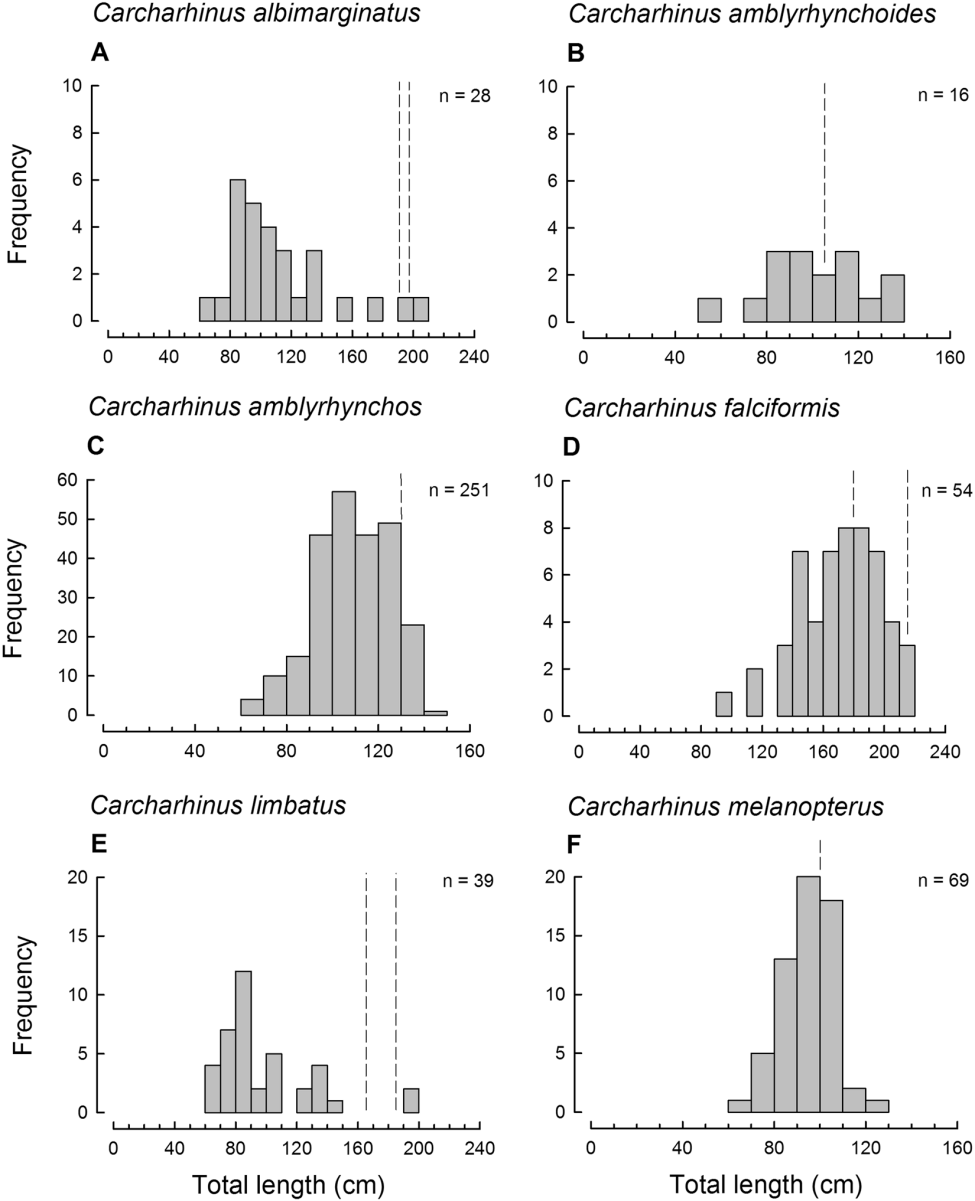
Length frequency histogram of six shark species represented by more than 10 individuals in the dried shark fin batch from the Milne Bay Province PNG. Total number (n) of sampled fins and length at maturity is given for each species (left dashed line denotes known length of maturity for males, right dotted line denotes known length at maturity for females; a single dashed line indicates that both sexes mature at that size; for length at maturity/species see Table 3 references).

**Figure 3:**
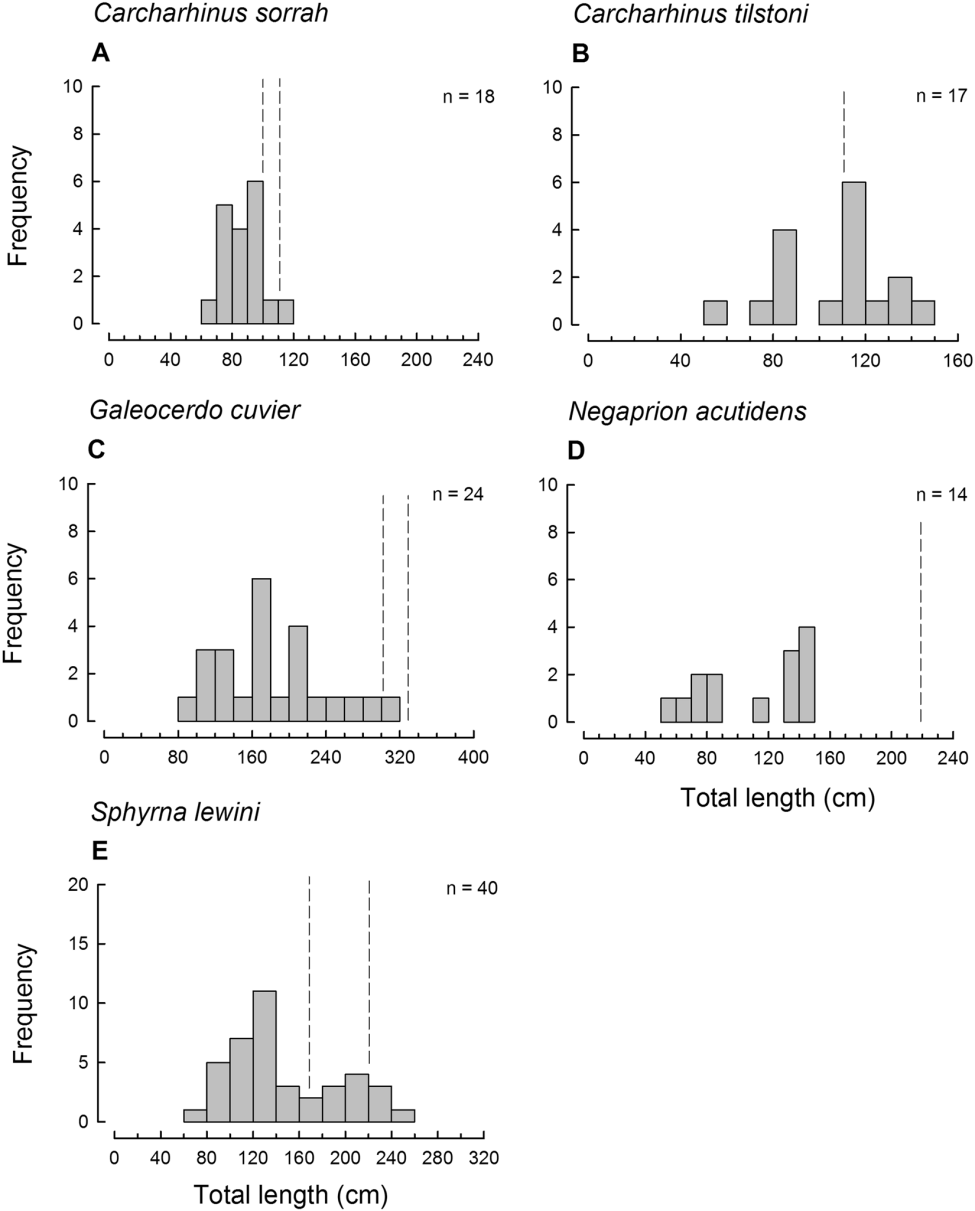
Length frequency histogram of five shark species represented by more than 10 individuals in the dried shark fin batch from the Milne Bay Province PNG. Total number (n) of sampled fins and length at maturity is given for each species (left dashed line denotes known length of maturity for males, right dotted line denotes known length at maturity for females; a single dashed line indicates that both sexes mature at that size; for length at maturity/species see Table 3 references).

The length frequency histograms for all species (represented by more than 10 individuals, see Figs 2 and 3) showed that a vast majority of sharks that contributed to this batch of artisanal sourced fins were immature. For all species, catches included individuals close to the size at birth, mostly through to the size at maturity. For some species, e.g., *C. albimarginatus*, *C. amblyrhynchos*, *C. limbatus*, *C. sorrah*, *G. cuvier* and *S*. *lewini*, only a small percentage of the individuals present in the catch were possibly adult. Furthermore, no adults of *N. acutidens* were recorded, with all 14 individuals much smaller than the known size at maturity for this species.

**Table 3:** Fin to length conversion and length to weight conversion with the source of the parameters used for each species (where n>1 individuals observed) recorded from the dried fins (where D1H = first dorsal fin height).

Species	Fin to total length conversions		Length to weight conversions		
	Estimated TL	Source	*a*	*b*	Source
*Hemipristis elongata*	=(D1H/0.115) + 1.666	^42, 43^, W.White unpubl. data	0.00162	3.21	^44^
*Carcharhinus albimarginatus*	=D1H/0.097	^45^	0.00201	3.23	^50^
*Carcharhinus altimus*	=(D1H/0.1005) − 0.4778	^47^, W.White unpubl. data	0.00189	3.23	^48^
*Carcharhinus amblyrhynchoides*	=(D1H/0.133) + 1.835	^47^, W.White unpubl. data	0.00265	3.21	^44^
*Carcharhinus amblyrhynchos*	=D1H/0.103	^45^	0.00746	2.98	^44^
*Carcharhinus amboinensis*	=D1H/0.122	^45^	0.00194	3.27	^44^
*Carcharhinus brevipinna*	=D1H/0.094	^45^	0.00113	3.33	^44^
*Carcharhinus falciformis*	=(D1H/0.088) + 1.616	^47^, W.White unpubl. data	0.00201	3.23	^50^
*Carcharhinus leucas*	=D1H/0.102	^45^	0.00271	3.2	^46^
*Carcharhinus limbatus*	=D1H/0.125	^45^	0.00251	3.125	^51^
*Carcharhinus melanopterus*	=D1H/0.102	^45^	0.00325	3.649	^52^
*Carcharhinus plumbeus*	=D1H/0.152	^48^	0.00142	3.31	^48^
*Carcharhinus sorrah*	=(D1H/0.106) + 1.523	^49^, W.White unpubl. data	0.00079	3.46	^53^
*Carcharhinus tilstoni*	=D1H/0.125	using *C. limbatus* conversion	0.00475	3.06	^53^
*Galeocerdo cuvier*	=D1H/0.084	^54^	0.00141	3.24	^55^
*Negaprion acutidens*	=(D1H/0.129) + 5.003	^54^, W.White unpubl. data	0.001208	3.29	^56^
*Sphyrna lewini*	=D1H/0.132	^54^	0.00399	3.03	^57^
*Sphyrna mokarran*	=D1H/0.166	^54^	0.00123	3.24	^57^
*Sphyrna zygaena*	=D1H/0.137	^54^	0.0126	2.81	^50^
*Anoxypristis cuspidata*	=D1A/0.103	W.White unpubl. data	0.005	2.474	^58^
*Rhynchobatus australiae*	=(D1H/0.109) + 0.516	W.White unpubl. data	0.004	3.0145	W. White unpubl. data
*Glaucostegus typus*	=(D1H/0.141) + 4.729	W.White unpubl. data	0.006	2.918	W. White unpubl. data

## Discussion

Our study is the first detailed examination of species composition based on DNA barcoding of elasmobranchs in an artisanal fishery of PNG. The identification of unknown elasmobranch fins from the PNG fin buyer, through a combination of fin images and genetic identifications enabled a very high degree of species identification. In our study and following unsuccessful attempts to use online shark fin identification resources, fin images (from either juveniles or adults) alone were not able to identify species. DNA barcoding was required when few other species-specific morphology/distinguishing characteristics were available. Our DNA COI barcoding successfully enabled delineation of the elasmobranchs observed in this survey (approximately 94% of the fins were identified to species level). For the remainder, a lack of preservation or poor storage conditions may have affected a small number of fins in the batch as bacterial contamination was noted (as evidenced from the sequencing). Additionally for some samples, while a PCR product was produced, the bi-directional sequencing did not pass quality control.

The COI sequencing successfully enabled the identification of the carcharhinids that had contributed to this batch of fins including *Carcharhinus limbatus*, *Carcharhinus tilstoni* and *Carcharhinus amblyrhynchoides*. These three species have previously been shown to be difficult to identify in the field and are often grouped together in a ‘blacktip shark complex’^19,34–36^. In our study, a relatively high level of species resolution between these three species was possible using mtDNA sequencing – particularly when multiple barcoding regions were examined. While *C. limbatus* and *C. tilstoni* have previously been shown in other studies to have low COI divergence^33,34^, in this study, the COI sequencing results (which delineated *C. limbatus*, *C. tilstoni* and *C. amblyrhynchoides*) were concordant with those from the ND2 gene. Furthermore, several fins that were morphologically identified in the field as being putative silvertip sharks (*Carcharhinus albimarginatus*), were genetically identified as belonging to grey reef sharks (*C. amblyrhynchos*) that had white tip fins. In addition to describing the species composition of the fins, our study also demonstrated that several species displayed intra-specific variation, albeit at low levels (e.g., *S. lewini*, *C. amblyrhynchos*, *C. altimus*). Given the use of the COI gene here for species identification, rather than a more variable gene fragment (e.g., mtDNA D-loop) for intra-specific genetic diversity assessment, this is not unexpected.

The Milne Bay Province is a data poor region of PNG, with little known about elasmobranch utilisation. Genetic identification of the dried fins sampled from the Asiapac fin buyer provided us with substantial baseline information as to the elasmobranch species caught in the region, and the sizes of animals caught in the artisanal fishery. Some of these species (e.g., *S. zygaena*) had not previously been recorded from these provincial artisanal waters. Nevertheless, the overall species composition represented in the fin batch was not surprising given the preferred habitats of the most frequently caught species (i.e., grey reef, blacktip reef, silky, blacktip, tiger and silvertip sharks and scalloped hammerheads) varies from shallow coastal and tropical reefs to waters over insular shelf areas – all of which are presumably accessible by the artisanal fishers in this province. Our study highlighted the medium to high diversity (with over 20 different species being detected) of elasmobranchs caught in the artisanal fishery with most of this information being considered new, as species catch rates in this artisanal fishery were not previously known or retained. In contrast, the high number of *C. amblyrhynchos* shark fins is not surprising given the predominantly coral reef habitats in this province. Although this could indicate that the population of *C. amblyrhynchos* within the provincial waters of Milne Bay is part of a relatively large number of individuals, dive operators in the area state they now observe far less *C. amblyrhynchos* than in previous decades. This species was also the most frequently caught shark species in a neighbouring Indonesian study^13^. Given the geographic closeness of PNG and Indonesia (and the numbers of grey reef sharks that have been caught), the analysis of both genetic and demographic connectivity of this species is highly important.

A relatively large number (20%) of the fins in this study came from taxa that are currently IUCN listed as threatened species (i.e., Vulnerable or Endangered). Furthermore, the estimate of 2 000 kg of *C. falciformis* (that contributed product to this single batch of fins) is despite a recent Conservation and Management Measure (CMM) for Silky Sharks 2013–08 (https://www.wcpfc.int/system/files/CMM%202013-08%20CMM%20for%20Silky%20Sharks_0.pdf) by the WCPFC. However as noted previously, while the CMM for silky sharks caused the subsequent closure of the large scale shark fishing in PNG, for small scale fishing/artisanal activities there are no national management arrangements (i.e., no regulations) in place^16^. Another significant finding from this study was that the majority of sharks recorded from the fin batch (i.e., *C. albimarginatus*, *C. amblyrhynchos*, *C. falciformis* and *S. lewini*) were also key species caught within the target longline fishery which operated prior to July 2014 (PNG NFA/CSIRO unpupl data). From a regional perspective, many of these fins come from species that are under worldwide pressure, with similarly concerning catch levels observed in neighbouring Indonesia and regional areas^3,8,13,21^. Additionally, following estimation of catches, extrapolated length frequencies and weight estimates, we identified a biological issue with individuals that are harvested from the area. As was found in Indonesia^13^, a large proportion of the elasmobranchs in the Milne Bay artisanal fishery are caught immature or before individuals have reached their length at maturity – these individuals are not reproducing. This could be an indication of unsustainable shark populations if the harvested animals are not contributing to the next generation.

Putting our results into context, this study is a snapshot of the artisanal shark fishery which provides shark fins to the Milne Bay fin buyers. Although we do not know the precise locations where these elasmobranchs have been caught (albeit the fins going to Asiapac are artisanal sourced), our results provide first records and detailed baseline information on the artisanal fishery in this area. While all fins examined were from the Milne Bay Province, we cannot be certain if the elasmobranchs detected here are resident or transient through these local PNG waters (as fins were sampled at the processor and not directly from fishing boats). Combining this with the uncertainty surrounding connectivity of species in the region, the extent to which the Milne Bay shark fin industry is impacting on these regional (and for several, internationally listed) elasmobranch species is unknown.

The shark resources in Milne Bay are currently considered open access, with no limit on how much shark can be harvested^16^. We would expect many of these individuals from the ‘local’ populations to be connected to nearby regional populations (such as those in Indonesia and Australia), particularly as studies on the shark industry in Indonesia^8,13^ highlighted catches of the same Vulnerable and Endangered species (e.g., *S. lewini*, *N. acutidens*). Given this, there needs to be some form of control and monitoring of the shark catch in Milne Bay Province in the near future^16^. Several input and output controls that could be considered (including allocation of community based catch allowances) have been outlined^16^. While it is beyond the scope of this current research to advocate for particular strategies or control measures, our research here further highlights a substantial biological socio-economic issue. Previous work has showed that production of shark fins is a key income source that supports the livelihood strategies of some local communities in the Milne Bay Province^16^. This is particularly the case for isolated, low-income, island communities that have few alternative income sources. Shark fins pose as an economically viable primary product given that they do not require refrigeration, are easily processed and transported^16^. Therefore any resource management intervention aimed at addressing the biological sustainability issues for elasmobranchs identified here has the potential to have localised, undesired socio-economic impacts.

Within this socio-economic framework, the question of how to improve resource management to achieve a sustainable shark fishery is one that warrants discussion - particularly given PNG’s international commitments. Such commitments include its Memorandum of Understanding with the IUCN signed in 2013 (https://www.iucn.org/content/png-and-iucn-seal-environment-partnership) under which the government of PNG is to secure and manage the important biodiversity resources of the country. Papua New Guinea’s commitments as a signatory to the convention on international trade CITES (https://cites.org/eng/cms/index.php/component/cp/country/PG) could also be better addressed. Currently, its’ elasmobranch data is aggregated and it is not possible to identify and or monitor the trade in shark parts belonging to listed species in Class Elasmobranchii (Pristidae spp - *A. cuspidata*, *S. lewini*, *S. mokarran*, *S. zygaena*). As shown in this study, fins from these four species were part of the 150 kg of dried fins that were barcoded.

Our research in linking genetic species identification of dried fins from this PNG artisanal fishery, to species compositions and catch extrapolations will contribute to the more sustainable use of elasmobranch resources in PNG, as country specific and regional fisheries assessments rely on accurate species identifications and catch effort data. Genetic testing/barcoding of shark products is the most accurate method (particularly when whole animals are not accessible) to obtain elasmobranch species identifications irrespective of origin. Nonetheless, genetic testing and barcoding comes at a cost and large scale barcoding of elasmobranch fins (and other tissue types) in PNG is not currently viable due to budget and capacity limitations in-country. Requiring artisanal fishers to land shark whole would enable identification at the point of landing or sale (rather than harvest), but for the fishers, this would be a difficult requirement to meet^16^, and may reduce the socio-economic viability^16^ of shark harvesting in the PNG artisanal sector. Therefore, improving cost-effective local data collection methods, such as recording (where known) the elasmobranch species at the point of catch/harvest, would allow for better catch data to be obtained and provide a better understanding of the dynamics of the fishery over the longer term. We recommend this should be undertaken to enable the collection of species specific data rather than aggregating all species product data to just ‘shark fin’. Species specific data is more valuable information for fisheries and conservation managers particularly regarding CITES listed taxa and species which share trans-country boundaries.

## Methods

### Processing of shark fins

In March 2016, the dried shark fins present at Asiapac (one of two licensed fish buyers in the Milne Bay Province) in the Provincial capital Alotau were examined (see Fig. 1^16^). Previous work^16,25^ indicated that the majority of the dried fin traded through the two licensed buyers in Alotau are sourced from the artisanal fishing sector within the province. This sector includes specialised and targeted artisanal harvesting of shark in some areas using longlines and handlines as well as non-targeted and opportunistic harvesting^16^. At the time of the study, approximately 150 kg of dried shark fin were available for examination. This was about one month’s worth of purchases from fishers within the Milne Bay Province, but does not necessarily reflect when the sharks were caught as fishers and small-scale buyers may store fins for several months before selling to a licensed buyer. Licensed buyers are required to maintain a record of the source location of fins from within the Province (down to LLG). However, once purchased and stored, there is no requirement to identify which fins have come from which LLG^16^. For this reason, locality of harvests within the province was not explored.

All suspected first dorsal fins were separated from the remaining fins, with a total of 640 fins isolated from the main batch. The height (D1H), length (D1L) and anterior margin (D1A) of each of these fins were measured^37^. Note in some circumstances, not all of these three measurements could be taken (e.g., if free rear tip was damaged), but in all cases at least one measurement was obtained. An image and a small piece of tissue was taken from the majority (n = 557) of the fins, with each of these fins allocated a unique label number to be included in all images and used as the tissue sample identifier. An image and tissue sample was not taken from all first dorsal fins identified as one of the following three species; blacktip reef shark *C. melanopterus*, silvertip shark *C. albimarginatus* and silky shark *C. falciformis* as these species could be accurately identified from the fins alone. All samples were tracked throughout the genetic laboratory analysis pipeline based on the unique label number.

### Confirmation of first dorsal fins

**Figure 4:**
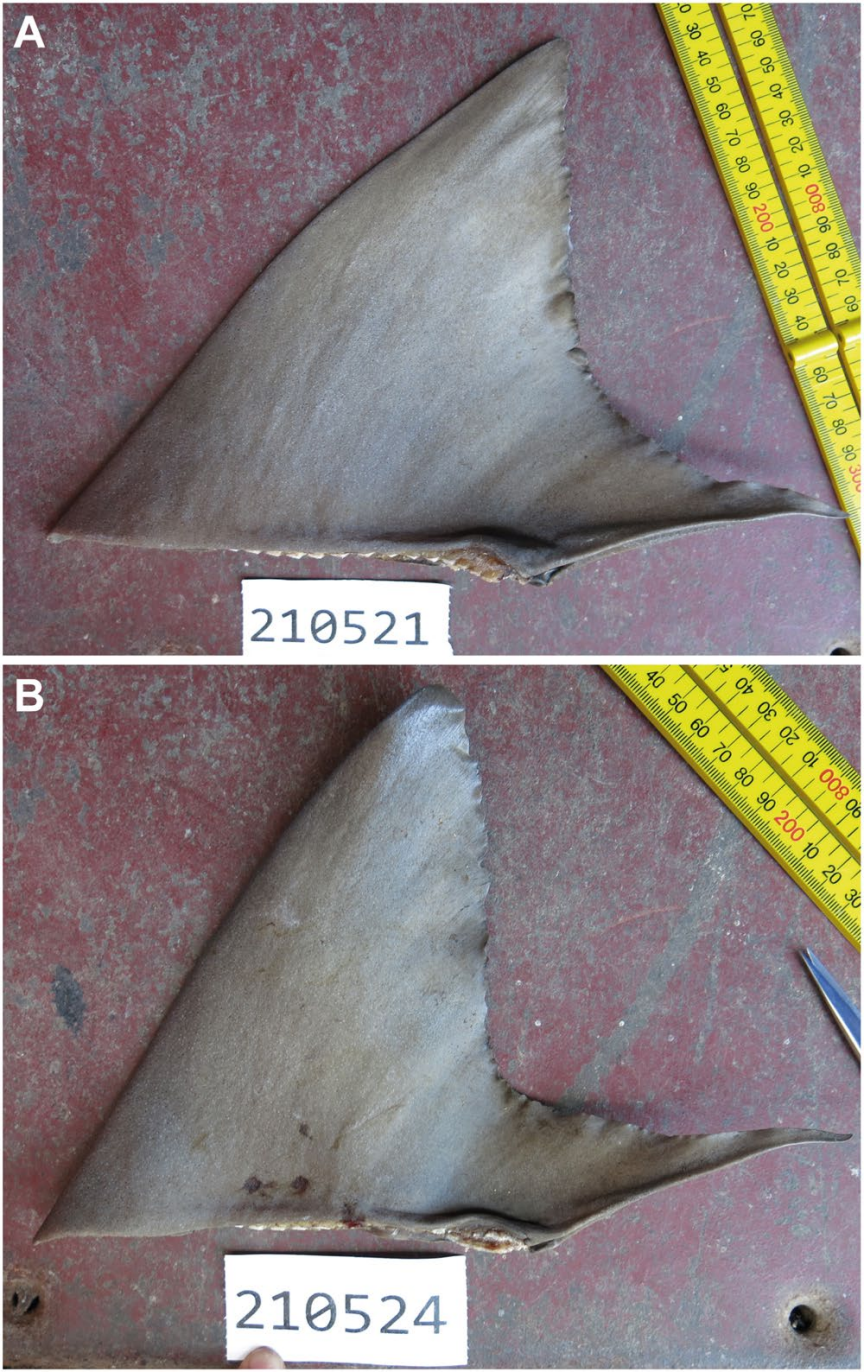
(**A**) First and (**B**) Second dorsal fins of *Negaprion acutidens*, highlighting the different morphology of the two fins, despite being similar in height (images ©Australian National Fish Collection).

Based on the genetic identification results (see later), all examined dorsal fins from species which possessed two similar-sized dorsal fins were reinvestigated. This was done to refine the number of individuals present in the dried fin batch and to eliminate double counting of individuals. The identified shark-like rays (i.e., narrow sawfish *A. cuspidata*, giant guitarfish *Glaucostegus typus* and whitespotted wedgefish *R. australiae*) possess similar-sized dorsal fins. For each of these species, the number of fins were sorted by their fin measurements and each pair working down the list was considered from a single individual with the larger of the pair allocated as the first dorsal fin and the smaller as the second dorsal fin. Thus, for *G. typus*, 14 fins were initially observed but this was considered to be from 7 individuals. For the sicklefin lemon shark *N. acutidens*, the first and second dorsal fins are similar in height but easily separable based on their morphology (Fig. 4), thus enabling the first dorsal fins to be separated out easily following species identification.

After second dorsal fins were eliminated from the batch of fins, a total of 623 individuals were confirmed from the 150 kg batch of dried fins examined. Images were stored in a database at CSIRO, while the fin clips were stored dried in 1.7 ml microfuge tubes at room temperature (while in the field) and were transferred to an ultra-low freezer (−80 °C) on arrival in the CSIRO marine genetics laboratory in Hobart.

### Genetic identification of fins

Total genomic DNA was extracted from approximately 25 mg fin tissue using the Promega Wizard® Genomic DNA Purification kit (Promega Corporation, USA) according to the manufacturer’s specification, with an overnight digestion step at 55 °C; DNA was precipitated in 160 *µ*l water. DNA was normalised to 10 ng/*µ*l with working stock stored at 4 °C and the bulk of the DNA stored at −80 °C.

Approximately 650 base pair (bp) segment of the 5′ end of the mtDNA COI gene was primarily amplified using the primers Fish-BCL-5′TCAACYAATCAYAAAGATATYGGCAC-3′ and Fish-BCH-5′ ACTTCYGGGTGRCCRAARAATCA-3′^38^. Where additional mtDNA information was required to confirm species validation, the NADH-2 primers ASNM 5′ AAC GCT TAG CTG TTA ATT AA 3′ and ILEM 5′-AAG GAG CAG TTT GAT AGA GT-3′^39^ were also utilised. PCR amplifications were carried out in an ABI 9600 thermocycler (Applied Biosystems™, USA) performed in 25 *µ*l reactions which consisted of 12.5 *µ*l of GoTaq Master Mix Green (Promega), 1 *µ*l Bovine Serum Albumin (Promega), 1.0 *µ*l of each 10 *µ*M primer, 7.5 *µ*l water and 2 *µ*l of template DNA. The PCR conditions consisted of 94 °C for 3 mins, then 35 cycles of 94 °C for 1 min, 50 °C/48 °C (for COI and ND2 respectively) for 1 min 30 s, 72 °C for 1 min and a final extension step of 72 °C for 10 min.

Amplified PCR products were cleaned using Agencourt AMPure XP magnetic particles (Beckmann Coulter Life Sciences, USA) with the quality and quantity of cleaned products checked using a Nanodrop 8000 spectrophotometer (Thermo Scientific, USA). Purified PCR products were labelled with the Big Dye Terminator v3.1 cycle sequencing ready reaction kit (Thermofisher, USA), cleaned using Agencourt CleanSEQ (Beckmann Coulter) magnetic particles and then bi-directionally sequenced at the CSIRO marine genetics laboratories on a 16 capillary ABI 3130XL DNA Autosequencer (Applied Biosystems™, USA). Forward and reverse sequences were trimmed, *denovo* assembled, sequences were checked by eye and then converted into consensus sequences using Geneious (Biomatters Ltd, New Zealand) vers R8.1.4. Consensus sequences for each sample were compared using the BOLD^32^ IDS tool and GenBank BLASTn (via an internal application in Geneious) to check the similarity of sample sequences against existing database sequences. Species identification was based on a percentage of sequence identity, with homology of ≥99% as the criterion used here for species confirmation. BOLD was primarily used for species identity based on the COI sequence, while both COI and ND2 sequences were compared in GenBank.

Following species confirmations, consensus sequences for all confirmed elasmobranch species were aligned in Geneious using a MUSCLE alignment. Aligned sequences were then exported into MEGA version 6.0^40^. The nucleotide composition and genetic distances between and among identified species were calculated using a Jukes-Cantor^41^ model with rate variation among sites modelled with a gamma distribution, and all positions containing gaps and missing data were eliminated. As this was not a phylogenetic study, we did not produce phenograms or phylogenetic trees. Representative COI sequences from each of the species identified from the fin samples have been deposited in GenBank (https://www.ncbi.nlm.nih.gov/genbank/; Accession Numbers MF508658 – MF508698).

### Conversion of confirmed fins to species total lengths and weights

Fin measurements and species conversions used to estimate the total length of all individuals of each of the species identified are provided in Table 3 (along with the source of the conversion data used). Additional fin measurements from individuals of known length were taken by one of us (WW) from whole specimens examined in the field and from preserved specimens housed in the CSIRO Australian National Fish Collection. First dorsal fin height (D1H) was used for the conversion to total length for all but one of the species, as a better range of data was available than for the other two fin measurements. The model for these conversions is:

$${\rm{W}}[\text{weight}]={\rm{a}}\times \text{TL}[\text{total}\,\text{length}{]}^{\wedge }{\rm{b}}$$

a and b parameters used to convert the estimated total lengths (cm) to total weight (g) are also provided in Table 1 together with their source.
