# Publisher Correction: Artisanal shark fishing in Milne Bay Province, Papua New Guinea: biomass estimation from genetically identified shark and ray fins

**DOI:** 10.1038/s41598-021-97067-z

**Published:** 2021-09-03

**Authors:** S. A. Appleyard, W. T. White, S. Vieira, B. Sabub

**Affiliations:** 1 CSIRO Australian National Fish Collection, National Research Collections Australia, Castray Esplanade, Hobart, 7001 Tasmania Australia; 2 CSIRO Oceans & Atmosphere, Castray Esplanade, Hobart, 7001 Tasmania Australia; 3 doMar Research, P.O. Box 223, Fremantle, 6959 Western Australia Australia; 4 Papua New Guinea National Fisheries Authority, P.O Box 2016, Port Moresby, National Capital District Papua, New Guinea, Australia

Correction to: *Scientific Reports* https://doi.org/10.1038/s41598-018-25101-8, published online 27 April 2018

The original version of this Article contained errors where References 42-58 cited in Table 3 were omitted from the Reference list. The References are listed below:

42. Smith, J.L.B. The rare shark *Hemipristis elongatus* (Klunzinger), 1871, from Zanzibar and Mozambique. *Ann. Mag. Nat. Hist*. **10,** 555–561 (1957).

43. Bass, A.J. Records of little-known sharks from Australian waters. *Proc. Linn. Soc. N.S.W.* **103,** 247–254 (1979).

44. Stevens, J.D. & McLoughlin, K.J. Distribution, size and sex composition, reproductive biology and diet of sharks from northern Australia. *Aust. J. Mar. Freshw Res*. **42,** 151–199 (1991).

45. Bass, A.J., D'Aubrey, J.D. & Kistnasamy, N. Sharks of the east coast of southern Africa. I. The genus Carcharhinus (Carcharhinidae). *Sth Afr. Assoc. Mar. Biol. Res., Ocean. Res. Inst*. **33,** 168 pp (1973).

46. Branstetter, S., Stiles, R. Age and growth estimates of the bull shark, *Carcharhinus leucas*, from the northern Gulf of Mexico. *Environ. Biol. Fish.* **20,** 169–181 (1987).

47. Garrick, J.A.F. Sharks of the genus Carcharhinus. *NOAA Tech. Rep. NMFS Circular* **445,** 1–194 (1982).

48. Joung, S. J., Liao, Y.Y. & Chen, C.T. Age and growth of sandbar shark, *Carcharhinus plumbeus*, in northeastern Taiwan waters. *Fish. Res.* **70,** 83-96 (2004).

49. Garrick, J.A.F. Additions to the revision of the shark genus Carcharhinus: synonymy of Aprionodon and Hypoprion, and description of a new species of Carcharhinus (Carcharhinidae). *NOAA Tech. Rep. NMFS* **34,** 1–26 (1985).

50. Branstetter, S. Age, growth and reproductive biology of the silky shark, *Carcharhinus falciformis*, and the scalloped hammerhead, *Sphryna lewini*, from the northwestern Gulf of Mexico. *Environ. Biol. Fish.* **19,** 161–173 (1987).

51. Castro, J.I. Biology of the blacktip shark, *Carcharhinus limbatus*, off the southeastern United States. *Bull. Mar. Sci*. **59**, 508–522 (1996).

52. Lyle, J.M. Observations on the biology of *Carcharhinus cautus* (Whitley), *C. melanopterus* (Quoy & Gaimard) and *C. fitzroyensis* (Whitley) from northern Australia. *Aust. J. Mar. Freshw. Res*. **38,** 701–710 (1987).

53. Stevens, J.D. & Wiley, P.D. Biology of two commercially important carcharhinid sharks from northern Australia. *Aust. J. Mar. Freshw Res*. 37, 671-688 (1986).

54. Bass, A. J., D'Aubrey, J.D., Kistnasamy, N. (1975). Sharks of the east coast of southern Africa. III. The families Carcharhinidae (excluding Mustelus and Carcharhinus) and Sphyrnidae. *Sth Afr. Assoc. Mar. Biol. Res., Ocean. Res. Inst*. **38,** 100 pp (1975).

55. Randall, J.E. Review of the biology of the tiger shark (*Galeocerdo cuvier*). *Aust. J. Mar. Freshw. Res*. **43,** 21–31 (1992).

56. Stevens, J.D. Biological observations on sharks caught by sport fishermen off New South Wales. *Aust. J. Mar. Freshw. Res*. **35,** 573–590 (1984).

57. Stevens, J.D. & Lyle, J.M. Biology of three hammerhead sharks (*Eusphyra blochii*, *Sphyrna mokarran* and *S. lewini*) from northern Australia. *Aust. J. Mar. Freshw Res*. **40,** 129–146 (1989).

58. Salini, J.P. *et al.* *Northern Australian sharks and rays: the sustainability of target and bycatch species*, phase 2, project no. 2002/064, Fisheries Research and Development Corporation report (2007).

The original Article has been corrected.

